# Photobiomodulatory Effects of Low-Power LED Light on Cultured Human Umbilical Vein Endothelial Cells

**DOI:** 10.3390/jcm14113959

**Published:** 2025-06-04

**Authors:** Ikuro Kato, Toshikatsu Suzumura, Yoshihiko Sugita, Satoshi Doi, Atsuo Komori, Yukinori Ueno, Yuki Ito, Seeta Kato, Waka Yoshida, Ryoko Kawai, Katsutoshi Kubo, Hatsuhiko Maeda

**Affiliations:** 1 Department of Oral Pathology/Forensic Odontology, School of Dentistry, Aichi Gakuin University, Nagoya 4648050, Japan; 2 Research Institute of Advanced Oral Science, Aichi Gakuin University, Nagoya 4648050, Japan

**Keywords:** photobiomodulation, LED light irradiation, endothelial cells

## Abstract

**Objective:** This study aimed to evaluate the photobiomodulatory (PBM) effects of low-power light-emitting diode (LED) irradiation on cultured human umbilical vein endothelial cells (HUVECs), focusing on changes in cellular metabolic activity and morphology. **Materials and Methods**: HUVECs were cultured and divided into three groups: control (no irradiation), red LED (655 nm), and blue LED (455 nm). Cells were irradiated once with a total energy dose of 4 J over 60 s. Cellular metabolic activity was assessed at 0, 1, 3, and 6 h post-irradiation using the WST-8 assay. Morphological changes were examined 3 h post-irradiation using rhodamine–phalloidin staining and confocal laser scanning microscopy. **Results**: Red LED irradiation significantly enhanced metabolic activity immediately and at 3 h post-irradiation compared to the control group. Blue LED irradiation showed a non-significant trend toward increased metabolic activity at 1 and 3 h. Morphometric analysis revealed increases in cell area, perimeter, and Feret diameter in both LED-irradiated groups, with more pronounced changes observed in the red LED group. **Conclusions**: Low-power red LED light (655 nm) effectively promotes metabolic activation and induces morphological changes in vascular endothelial cells, suggesting its potential application in angiogenesis and wound healing. Due to its safety and accessibility, LED-based PBM may serve as a promising therapeutic modality for soft tissue regeneration in both clinical and home-care settings.

## 1. Introduction

Photobiomodulation (PBM) therapy, also known as low-level laser therapy (LLLT), has recently gained attention as a non-invasive treatment that utilizes light energy to enhance or modulate cellular activity, thereby improving or restoring tissue function. PBM has been widely applied in the treatment of skin lesions, neurodegenerative diseases, pain management, wound healing, and tissue regeneration [1,2,3]. PBM elicits biological responses through the application of low-intensity laser or light-emitting diode (LED) irradiation, producing photochemical effects that support wound healing, anti-inflammatory and analgesic effects, peripheral nerve activation, and improved circulation [4,5]. While most PBM studies to date have employed laser devices, research on the use of LED light for PBM remains limited [6]. The vascular system plays a critical role in delivering oxygen and nutrients to tissues while removing carbon dioxide and metabolic waste, thereby maintaining organ function. In addition, blood vessels form inter-organ networks that regulate homeostasis via signaling molecules. The vasculature also plays a pivotal role in wound healing [7]. Vascular endothelial cells form the innermost layer of blood vessels and are vital for vascular integrity. They secrete vasoactive substances such as nitric oxide (NO) and endothelin, and they participate in vascular tone regulation, leukocyte adhesion, vascular permeability, and the coagulation–fibrinolysis balance [7]. Although these cells divide infrequently—approximately once every 100 to 1000 days—they are capable of rapid proliferation in response to tissue injury. They also release factors that regulate coagulation, mediate platelet aggregation, and promote angiogenesis [8]. The term “angiocrine signals” has recently been used to describe molecules secreted by endothelial cells that influence tissue remodeling [7]. Previous PBM studies using laser irradiation on endothelial cells have demonstrated enhanced cellular metabolic activity and proliferation [9,10,11]. While clinical laser devices require strict safety management and regulation, LED devices do not require such classifications. This makes LED-based PBM more accessible to a broader range of healthcare professionals, including those in home-care settings. It also suggests the potential for LED light to be used in treating soft tissue conditions, particularly in elderly populations. In the present study, we investigated the effects of red and blue low-level LED irradiation on cultured human umbilical vein endothelial cells (HUVECs) to assess the photobiomodulatory effects on endothelial cell metabolism and morphology. In addition to their key roles in vascular integrity and tissue remodeling, endothelial cells have recently been recognized as regulators of angiocrine signaling, influencing tissue regeneration and repair. Given the rising interest in photobiomodulation (PBM) as a non-invasive strategy for enhancing cellular performance, further clarification of LED effects on endothelial function could contribute to novel therapeutic approaches in wound healing and angiogenesis.

## 2. Materials and Methods

### 2.1. Cell Culture

Normal human umbilical vein endothelial cells (HUVECs; KE-4109; KURABO, Osaka, Japan) were used in this study. The cells were cultured at 37 °C in a 5% CO_2_ incubator, with medium changes every three days. The culture medium used was HuMedia-EB2 (KURABO, Osaka, Japan), supplemented with glucose (1 mL, 3.00 M), arginine (1 mL, 0.36 M), fetal bovine serum (FBS; 10 mL, 100% *v*/*v*), hydrocortisone (0.5 mL, 1.34 mg/mL), heparin (0.5 mL, 10 mg/mL), and antibiotics (gentamicin and amphotericin B; 0.5 mL, 50 mg/mL and 50 µg/mL, respectively). To promote proliferation, additional supplements included human recombinant epidermal growth factor (hEGF; 0.5 mL, 10 µg/mL) and human recombinant basic fibroblast growth factor (hFGF-B; 0.5 mL, 5 µg/mL). An overview of these components is presented in Table 1. Cells were subcultured upon reaching 80% confluency. For experiments, cells were seeded in 12-well plates at a density of 3 × 10^4^ cells/mL after reaching confluency again post-passaging.

### 2.2. LED Light Irradiation Conditions

**Figure 1:**
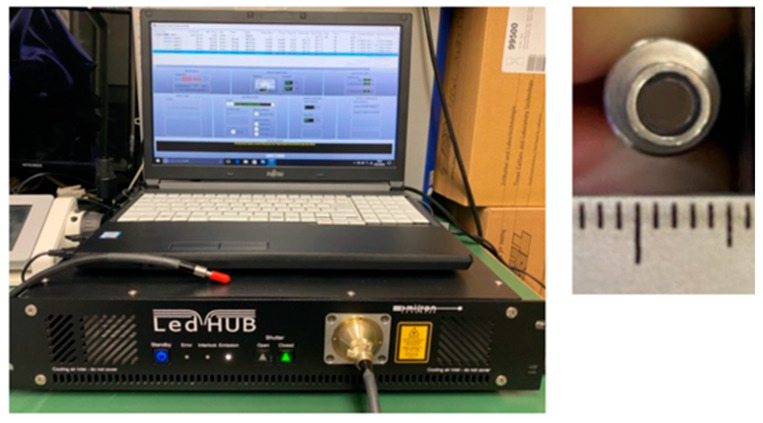
LED light source and control system. The LED Hub and control PC used in this study. The probe tip diameter is approximately 4 mm.

**Figure 2:**
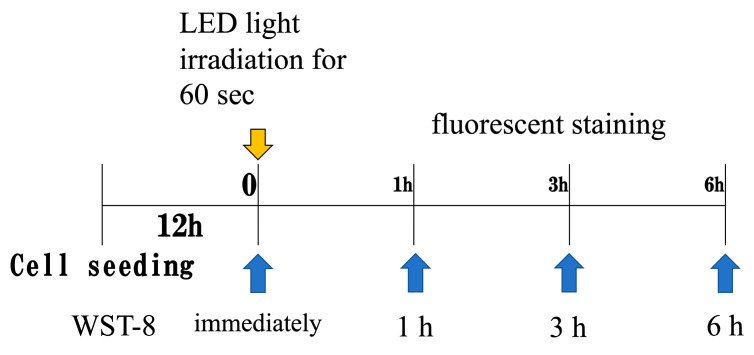
LED light irradiation conditions. Irradiation was performed 12 h after seeding cells into 12-well plates. Metabolic activity was assessed at 0, 1, and 3 h post-irradiation.

**Figure 3:**
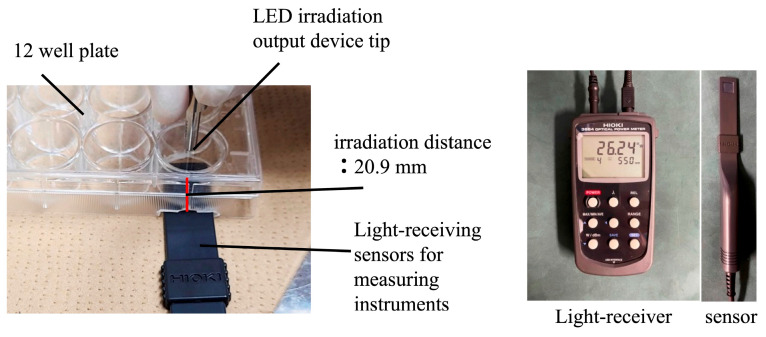
Measurement devices. Power detector and light power meter used to verify irradiation output and intensity.

HUVECs were assigned to three groups: control (no irradiation), red LED (655 nm), and blue LED (455 nm) *(n* = 4 per group). The control group was cultured without light exposure. Twelve hours after seeding into 12-well plates, the culture medium was replaced, and LED irradiation was performed. The LED light source used was the LED Hub (Omicron, Rodgau, Germany) with a 3 mm diameter probe tip (Figure 1). Irradiation parameters were as follows: 4 J total energy, 67 mW irradiance, 20.9 mm irradiation distance, and 60 s exposure duration. No temperature increase due to irradiation was observed, as confirmed by a non-contact thermometer (Figure 2). Output and irradiance were verified using a power detector and light power meter (Model 3664, Hioki Electric Co., Ltd., Nagano, Japan) (Figure 3). The 655 nm red LED was selected for its well-established ability to activate mitochondrial cytochrome c oxidase (CCO), promoting ATP synthesis and enhancing cellular metabolism. The 455 nm blue LED was chosen to explore possible effects mediated by opsin-related signaling pathways, which have been implicated in blue light-induced responses in other cell types such as keratinocytes and fibroblasts [12]. A single irradiation was employed to isolate the acute cellular effects of LED exposure without confounding by cumulative stimulation. This design enables clearer interpretation of early-stage cellular responses.

### 2.3. Assessment of Cellular Metabolic Activity

Cellular metabolic activity was assessed at 0, 1, 3, and 6 h after LED irradiation using the Cell Counting Kit-8 (WST-8; Dojindo Laboratories, Kumamoto, Japan). After removing the culture medium, cells were washed three times with PBS, and fresh medium was added to each well, followed by WST-8 reagent. The cells were incubated for 1 h. Subsequently, the supernatant was transferred to a 96-well plate, and absorbance was measured at 450 nm using a microplate reader. For metabolic and morphological assessment, medium without proliferation supplements was used.

### 2.4. Morphological Evaluation by Fluorescence Staining

**Figure 4:**
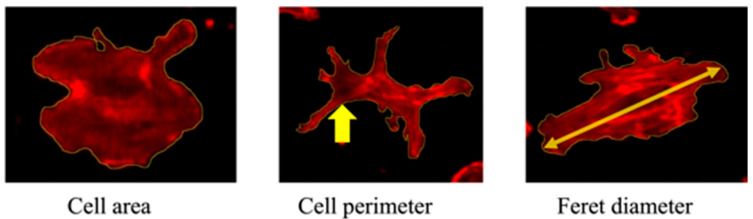
Cell morphology parameters. Representative images showing measurements of the cell area, perimeter (indicated by arrow), and Feret diameter (arrow).

Morphological assessment was conducted 3 h after LED irradiation using fluorescence staining. Cells were fixed in formalin and stained with Rhodamine Phalloidin to visualize the cytoskeleton. Imaging was performed using a confocal laser scanning microscope. Morphometric analysis using ImageJ 1.52q (NIH, Bethesda, MD, USA) involved manually setting intensity thresholds to delineate cell boundaries. Overlapping cells and debris were excluded based on visual inspection. A minimum of 50 individual cells per group were analyzed, and all image processing was conducted by two independent investigators blinded to the group assignments. Sterile cover glasses were placed in 6-well plates, and cells were seeded at a density of 3 × 10^4^ cells/mL. After 12 h, cells were irradiated with LED light. At 3 h post-irradiation, the medium was removed and the cells fixed with 10% formalin. Following PBS washes, cells were stained with Rhodamine Phalloidin (Thermo Fisher Scientific, Waltham, MA, USA) diluted 1:100 in 1% BSA in PBS. After 30 min, the stain was removed, and the cells were washed with distilled water before being mounted using Fluoromount (Cosmo Bio, Tokyo, Japan). Cell morphology, including area, perimeter, and Feret diameter, was analyzed (Figure 4).

### 2.5. Statistical Analysis

Statistical comparisons among the groups were performed using one-way analysis of variance (ANOVA), followed by Tukey’s multiple comparison test. A *p*-value of less than 0.05 was considered statistically significant. The sample size *(n* = 4 per group) was determined with reference to similar exploratory studies involving photobiomodulation in endothelial cells. Although a formal power analysis was not conducted, this pilot-scale design provides a basis for future validation studies with larger sample sizes and statistical power calculations.

### 2.6. Ethical Statement

The cells were purchased from KURABO Co., Ltd. (Product Code: KE-4109), a certified commercial supplier, and were handled in accordance with the ethical standards of the Declaration of Helsinki. The supplier confirmed that the cells were collected with donor consent and anonymized prior to distribution. As such, the Ethics Committee of Aichi Gakuin University determined that individual informed consent was not required for this study.

## 3. Results

### 3.1. Cellular Metabolic Activity

In the red LED irradiation group, cellular metabolic activity was significantly higher than the control group immediately and 3 h after irradiation. A trend of increased metabolic activity was observed 1 h post-irradiation, but it was not statistically significant. By 6 h post-irradiation, metabolic activity was comparable to the control group.

In the blue LED irradiation group, metabolic activity was similar to the control group immediately after irradiation but showed a trend of increase at 1 and 3 h post-irradiation. Although a trend toward increased metabolic activity was observed in the blue LED group at 1 and 3 h post-irradiation, these differences did not reach statistical significance (*p* > 0.05) and should be interpreted with caution as preliminary findings. By 6 h post-irradiation, metabolic activity tended to be lower than the control group.

**Figure 5:**
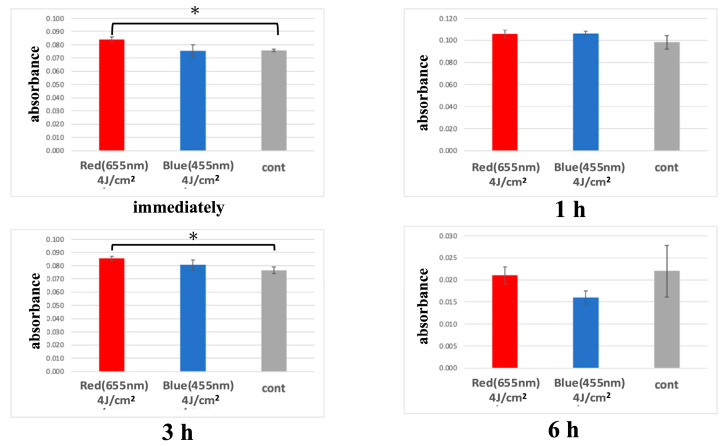
Metabolic activity measured by WST-8 assay at 0, 1, and 3 h after LED irradiation, based on absorbance at 450 nm. (* *p* < 0.05).

Additionally, the red LED group exhibited higher metabolic activity compared to the blue LED group immediately and 3 h post-irradiation, though this difference was not significant. At 6 h post-irradiation, the red LED group showed a tendency for higher metabolic activity compared to the blue LED group (Figure 5).

### 3.2. Comparison of Confocal Laser Microscopy Images 3 Hours After LED Irradiation

**Figure 6:**
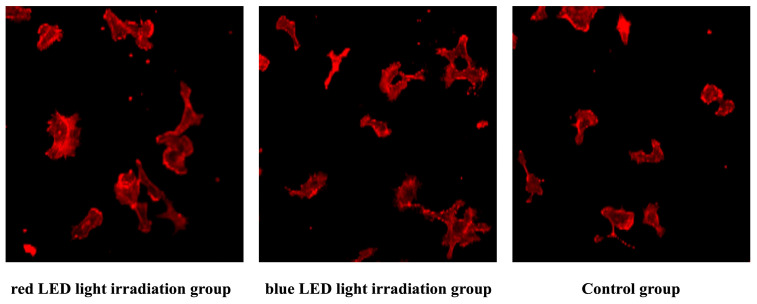
Fluorescence-stained images of HUVECs. Representative images of cells stained with Rhodamine Phalloidin to visualize the actin cytoskeleton.

**Figure 7:**
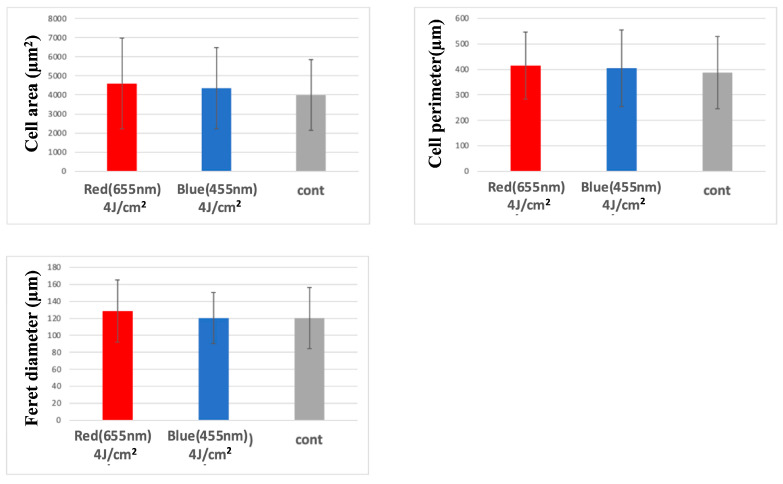
Morphological comparison via confocal microscopy at 3 h post-irradiation. Both red and blue LED irradiation groups showed increased cell size parameters compared to the control group.

Despite observed increases in cell area, perimeter, and Feret diameter following LED irradiation, none of these morphological differences reached statistical significance. Thus, conclusions regarding photobiomodulatory effects on cell morphology remain speculative and require further investigation (Figure 6 and Figure 7).

## 4. Discussion

Wound healing involves complex processes such as the infiltration of inflammatory cells and the formation of granulation tissue, both of which depend on the activation and proliferation of vascular endothelial cells. HUVECs, used in the present study, are widely employed as a representative model for endothelial cell behavior in research on atherosclerosis, angiogenesis, and inflammation [13,14,15,16,17]. Although HUVECs are widely used as a model for vascular endothelium, they differ from microvascular endothelial cells typically found in wound beds. Moreover, static in vitro conditions do not replicate the shear stress experienced in vivo. In this study, a in vitro culture was intentionally selected to isolate the photobiological effects of LED irradiation under well-controlled experimental conditions. This study investigated the photobiomodulatory (PBM) effects of low-power LED irradiation on HUVECs, which play a vital role in vascular function and tissue repair. PBM therapy typically employs red (500–700 nm) or near-infrared (700–1400 nm) wavelengths. Initially developed using ruby lasers (694 nm) in the late 1960s, PBM—formerly known as low-level laser therapy (LLLT)—has been used primarily for promoting wound healing, alleviating pain, and reducing inflammation [18]. While laser light was predominantly used until the early 2000s, recent years have seen increasing adoption of LED light as a PBM modality. Compared to laser devices, LED systems offer several advantages: a lower risk of thermal or ocular damage, no requirement for designated controlled environments, broader irradiation coverage, lower cost, and greater ease of use. These characteristics have expanded the potential applications of LED-based PBM, particularly in home-care and non-dental medical settings [4,5]. The red and near-infrared wavelengths commonly used in PBM are known to penetrate tissues more effectively and are absorbed by cytochrome c oxidase in the mitochondria. Activation of this enzyme promotes electron transport, leading to increased ATP synthesis, generation of reactive oxygen species (ROS), and the activation of signaling pathways involved in cell proliferation, anti-inflammatory responses, and tissue regeneration [19,20].

Granulation tissue formation—a critical phase of wound healing—involves extracellular matrix production, fibroblast proliferation, and angiogenesis. PBM has been shown to enhance fibroblast activity and migration. For instance, irradiation with GaAlAs diode lasers at 830 nm, as well as with wavelengths of 670, 780, 692, and 786 nm, has been reported to promote fibroblast proliferation [21,22].

In addition to fibroblasts, endothelial cells also respond to PBM. Studies have shown that laser irradiation at 808 nm and longer near-infrared wavelengths (1064 and 1270 nm) at 10 mW/cm^2^ enhances nitric oxide (NO) release from endothelial cells [23]. NO plays a key role in vasodilation, and its reduction is associated with hypertension and atherosclerosis. The NO response has been reported to diminish when mitochondrial respiration is inhibited, suggesting that PBM-induced NO release is mitochondria-dependent [23]. Other studies have demonstrated that PBM (808 nm, 350 mW/cm^2^, 2 min/day for 7 days) can reverse cellular senescence markers and DNA damage in brain-derived endothelial cell lines, likely via the upregulation of endothelial nitric oxide synthase (eNOS) [24].

Consistent with these reports, our study found that a single irradiation with 655 nm red LED light significantly increased metabolic activity in HUVECs, particularly immediately and 3 h post-irradiation. However, the enhancement was transient, with no significant differences observed at 6 h post-irradiation. This suggests that cytochrome c oxidase activation and ATP production induced by red LED light may be short-lived.

Interestingly, a previous study using bone marrow-derived osteoblast-like cells showed that intermittent irradiation (every 1.5 h) with a total energy of 5.6 J more effectively enhanced metabolic activity than continuous exposure [25]. Although this study involved osteoblast-like cells, it raises the possibility that multiple or interval-based irradiation protocols may also benefit endothelial cells—an area worth exploring in future research.

Blue light (430–490 nm) has also been reported to elicit cellular responses via opsin receptor activation [26]. Opsins, photoreceptors expressed in various cell types, have been shown to regulate metabolic activity. For example, 488 nm blue light increases opsin 4 expression in keratinocytes, melanocytes, and fibroblasts, promoting proliferation and differentiation [27].

In our study, blue LED irradiation (455 nm) similarly showed a trend toward increased metabolic activity in HUVECs, although the effect was less pronounced than with red light. The difference may reflect wavelength-specific sensitivity of receptors or differences in tissue penetration, as red light generally penetrates more deeply than blue light and may exert stronger effects on endothelial cells in granulation tissue. Confocal microscopy revealed that both red and blue LED irradiation increased cell area, perimeter, and Feret diameter in HUVECs, with the red LED group exhibiting slightly greater morphological changes. Although the differences were not statistically significant, the trend supports the notion that PBM can influence not only cellular metabolism but also cell morphology. We hypothesize that opsins such as OPN3 and OPN4 may mediate blue light responses, based on evidence from keratinocytes and fibroblasts, though their expression in HUVECs remains unconfirmed. Although opsin-mediated phototransduction has been implicated in blue light responses, it remains unclear whether HUVECs endogenously express opsins such as OPN3 or OPN4. This uncertainty limits our ability to interpret the precise molecular mechanisms underlying blue light effects [28].

Clinically, LED-based PBM has been applied using both transcutaneous [29] and transmucosal [30] approaches. Red and near-infrared light can penetrate tissue to stimulate lymphocyte activation, anti-inflammatory cytokine release, and NO production [31]. However, scientific evidence for these effects remains limited, and standardized treatment protocols are still under development, particularly in animal and human models [32,33,34]. Recent clinical trials involving transcutaneous irradiation of the radial artery (660 nm, 100 mW, 30 min per session, 10 sessions) have shown improvements in pain relief, functional recovery, and mental health [29]. However, the underlying mechanisms remain unclear, and it is unknown whether the observed effects are attributable to leukocytes, erythrocytes, endothelial cells, or pericytes. To advance clinical applications of PBM, future studies should focus on optimizing irradiation parameters and elucidating the molecular and cellular mechanisms through in vitro and in vivo models.

The small sample size (*n* = four per group) limits the statistical power of this study. Therefore, the findings should be interpreted as preliminary, warranting validation in larger-scale studies. Future studies should not only assess metabolic and morphological changes but also incorporate functional endpoints such as migration, tube formation, and reactive oxygen species generation. Furthermore, the integration of co-culture systems or in vivo models will be essential to evaluate the translational value of LED-PBM for clinical wound management.

## 5. Conclusions

In conclusion, this study demonstrated that low-power red LED light (655 nm) promotes metabolic activity and induces morphological changes in cultured HUVECs, indicating a potentially relevant PBM effect compared to blue LED light (455 nm), although further in vivo validation is required. While these in vitro results suggest a potential for LED-based PBM in enhancing endothelial cell function, further validation using in vivo models is essential before clinical applications can be considered.

## Figures and Tables

**Table 1:** Composition of the culture medium. Detailed components used for HUVEC culture, including supplements for cell proliferation.

HuMedia-EG2 Reagents for Cell Proliferation	
basal medium	500 mL
glucose	1 mL (3.00 M)
arginine	1 mL (0.36 M)
fetal bovine serum (FBS)	10 mL (100% *V*/*V*)
hydrocortisone	0.5 mL (1.34 mg/mL)
heparin	0.5 mL (10 mg/mL)
gentamicin and amphotericin B	0.5 mL (gentamicin: 50 mg/mL; amphotericin B: 50 µg/mL)
human recombinant epidermal growth factor (hEGF)	0.5 mL (10 µg/mL)
human recombinant basic fibroblast growth factor (hFGF)	0.5 mL (5 µg/mL)

## Data Availability

Data are contained within the article.
